# Evaluation of DNA Extraction Methods for Microbial Community Profiling in Deadwood Decomposition

**DOI:** 10.1002/mbo3.70007

**Published:** 2024-11-13

**Authors:** Yanmei Zhang, Zewei Song, Jonathan S. Schilling

**Affiliations:** ^1^ Department of Plant and Microbial Biology University of Minnesota Saint Paul Minnesota USA; ^2^ BGI Research Sanya China

**Keywords:** amplicon, decomposer, DNA extraction, endophyte, metagenomics, wood

## Abstract

As technologies advance alongside metabarcoding and metagenomic resources, particularly for larger fungal genomes, DNA extraction methods must be optimized to meet higher thresholds, especially from complex environmental substrates. This study focused on extracting fungal genomic compounds from woody substrates, a challenge due to the embedment of endophytic and saprotrophic fungi within wood cells, the physical recalcitrance of wood, the adsorption of nucleic acids to wood polymers, and the release of downstream inhibitors. Hypothesizing that cetyltrimethylammonium bromide would be the best option, we compared prominent methods by extracting and sequencing microbial DNA from sound and decayed birch (*Betula papyrifera*) and pine (*Pinus resinosa*). DNA quantities varied significantly depending on extraction methods and decay stage. The quality of DNA, in terms of purity and integrity, significantly impacted whether the samples could be amplified and sequenced. However, amplicon sequencing of bacterial and fungal communities revealed no significant extraction bias. This, along with the sequencing effectiveness and cost/time efficiency, indicates that Qiagen is the gold standard for woody substrates. This study increases confidence in published amplicon data sets regardless of the extraction methods, provides a cost‐benefit table for making protocol decisions, and offers guidance on fungal DNA extractions from complex organic substrates (sound and decayed wood) that would best suit future metagenomic efforts.

## Graphical abstract

By extracting DNA from sound and decayed wood samples at various stages of natural decomposition in both conifer (gymnosperm) and angiosperm wood substrates, we found that DNA quality significantly impacted the ability to amplify and sequence the samples. Despite variability in extraction efficiency, extraction bias (the types of microbes identified) among the methods tested was negligible, lending confidence to comparisons of previously published amplicon data sets. This study provides a cost‐benefit table for making protocol decisions and offers novel guidance on fungal DNA extractions from complex organic substrates, which will benefit future metagenomic efforts currently challenging for fungi.

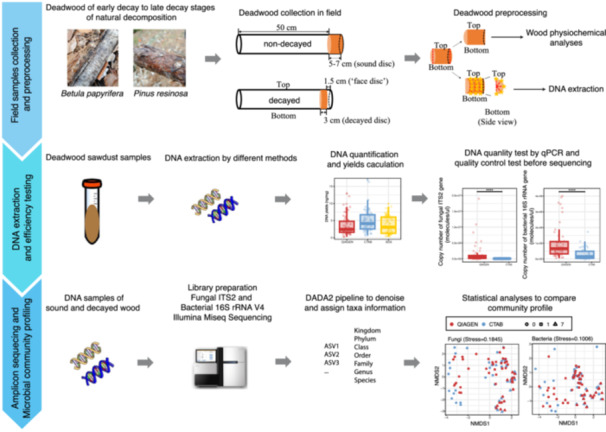

## 1 Introduction

DNA‐ and RNA‐based profiling methods to interrogate microbial communities and their ecological functions have been used for environmental samples of many different origins, enabling the detection of low‐abundance taxa or unknown microorganisms and offering more comprehensive insights into ecological patterns and strategies of microbial communities in various ecosystems (Fuhrman 2009; Escobar‐Zepeda, Vera‐Ponce De León, and Sanchez‐Flores 2015; Taş et al. 2021). Whole community fingerprinting, including the evolving classifications accuracy of “next‐generation” sequencing and “long‐read” sequencing (Tedersoo et al. 2021; Satam et al. 2023), relies on high‐quality DNA suitable for library preparation followed by sequencing (Endrullat et al. 2016; Bag et al. 2016; Costa et al. 2021). Community profiling by amplicon sequencing or shotgun sequencing has been commonplace for bacterial communities and is of increasing interest for larger‐genome fungal communities (Escobar‐Zepeda, Vera‐Ponce De León, and Sanchez‐Flores 2015; Quince et al. 2017; Donovan et al. 2018; Nilsson et al. 2019). To obtain suitable DNA, methods must be evaluated and optimized regarding their yields, integrity, and purity. They must also be tested for any extraction bias on sequencing results, an issue well‐known from some environmental samples, such as soil, water, animal guts, and so forth (Lombard et al. 2011; Demkina et al. 2023; Yuan et al. 2012; Costea et al. 2017).

Part of our justification for testing wood (sound and decayed) in this study, beyond our familiarity with the substrate, was its global importance in nutrient cycling and the related increase in amplicon and metagenomics efforts (Tláskal et al. 2021b). Wood decomposition redistributes carbon and other elements (e.g., nitrogen and calcium) from one of Earth's largest organic pools (Houghton 2007; Pan et al. 2011), and the biotic component of this process is mainly governed by fungal and bacterial communities (Hoppe et al. 2014; Johnston, Boddy, and Weightman 2016; Tláskal et al. 2021a; Haq et al. 2022). These biotic dynamics have proven hard to predict. Decomposers, particularly fungi, compete to dominate wood substrates, and their mechanisms are not functionally redundant. The fraction of carbon respired from wood‐bound carbon varies among fungi with distinct nutritional modes (Schilling et al. 2015, 2020), with some releasing more C to the atmosphere as CO_2_ (Rinne‐Garmston et al. 2019) and others shunting more C into soil organic matter or directly into microbial biomass (Magnússon et al. 2016). Predicting rates and fates for mineralized wood has been challenging (Cornwell et al. 2009; Šamonil et al. 2020), and sporocarp assessments fall short due to variability in production by species (Kubartová et al. 2012; Ovaskainen et al. 2013), life cycle timing/stage (Halme and Kotiaho 2012; Tomao et al. 2020), and environmental conditions (Kauserud et al. 2008). Predicting wood decomposition using DNA assessments and fungal molecular “traits” has thus emerged as a goal for community assembly studies (Fukami et al. 2010; Song et al. 2017; Cline et al. 2018) and for Earth Systems modeling (Keenan et al. 2013).

Our other justification for sampling sound and decayed wood was that it presented one of the more challenging substrates for DNA extraction (Asif and Cannon 2005). In the context of sequencing, high‐quality DNA is characterized as DNA that is predominantly of a high molecular weight without contaminating substances such as polysaccharides or phenolics that impede or inhibit DNA library preparation. Due to the rigid wood cell wall, wood dryness, and the presence of high quantities of inhibitors in wood, such as polysaccharides, polyphenols, and lignin compounds (Verbylaitė et al. 2010; Fatima et al. 2018), DNA isolation from sound wood (heartwood and sapwood), particularly for filamentous fungi that create a web of hyphae within wood cell lumens (Schwarze 2007), can be difficult. The inhibitory compounds are released with a shifting profile alongside deadwood composition, which presents additional challenges for microbial DNA extraction from decayed samples. Methods with cetyltrimethylammonium bromide (CTAB) or sodium dodecyl sulfate (SDS) have often been used to overcome some of these challenges. These methods involve using a surfactant to disrupt membranes, followed by organic extraction, selective DNA precipitation, and adding polyvinylpyrrolidone (PVP) to contend with phenolics and polysaccharides in wood (Jiao et al. 2012; Fatima et al. 2018). A less laborious alternative, the DNeasy PowerLyzer PowerSoil Kit (QIAGEN LLC, Germantown, MD, USA) has been increasingly accepted for sampling wood, relying on a spin column with a DNA‐binding membrane and a buffer system for cell lysis, DNA binding, and elution. It offers an easier approach that is widely accepted for sampling wood (Kielak et al. 2016; Makipaa et al. 2017; Lear et al. 2018). However, it has been unclear if these approaches yield similar or reliable results when substrate chemistries shift.

Given these justifications, this study aimed to compare the efficacy and potential extraction bias among three of the most commonly used methods to extract microbial DNA from wood (CTAB, SDS, Qiagen). To do this, we extracted DNA from sound and decayed wood from birch (*Betula papyrifera*) or pine (*Pinus resinosa*) at different decay stages (up to 7 years; decay stage V). We then tested DNA quantity and quality using multiple methods, and we assessed extraction biases using the Illumina MiSeq platform to sequence 16S ribosomal RNA (rRNA) V4 and internal transcribed spacer (ITS) region ITS2 and to compare species presence/absence and relative abundances among the extraction treatments.

## 2 Materials and Methods

### 2.1 Field Sampling and Preparation

**Figure A1:**
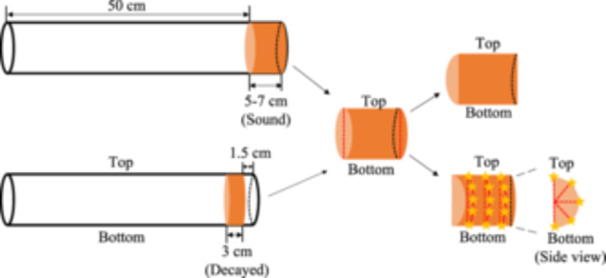
Log samplings in the field experiment and drill positions designed for DNA extraction. The red dotted lines indicated the cutting position when processing a whole disc into two half discs by evenly dividing the top and bottom. The yellow stars illustrated the drilling positions along the length of each half‐wood disc. A total of 15 holes were drilled per wood disc.

Sound and decayed wood were obtained from a long‐term field experiment at the Cloquet Experimental Forest Center, located in Cloquet, north Minnesota (46°42′08″ N, 92°32′53″ W). The forest plot sourcing the wood is dominated by paper birch (*B. papyrifera*), red pine (*P. resinosa*), and white spruce (*Picea glauca*). Ten healthy paper birch trees and 10 healthy red pine trees (7–9 cm diameter at the base, > 2.5 m height) were felled in October 2010. After stripping the branches, a 50‐cm stem section (“log”) and an additional 5–7‐cm stem section (“disc”) were cut out from near the base of each tree using a sterilized handsaw (Figure A1). Altogether, 20 discs were cut and immediately frozen at −80°C as time 0 samples (year 0; sound wood). The 20 logs were put in two E/W transects, with each log pointing N/S along the forest floor, to allow decay to develop in the field. In 2011 (year 1) and 2017 (year 7), a disc of ~3 cm was cut from one end of each log using a sterile handsaw after removing a “face” disc of ~1.5 cm, and all the discs were immediately frozen at −80°C.

In total, we used 60 discs collected in this trial, representing different decay stages for two tree species. After removing from −80°C, each frozen disc was sterilized by wiping with 70% ethanol, and the bark was then carefully removed using a sterile scalpel. The frozen disc was then split evenly into half by dividing the top and bottom with a hammer and chisel, with one half for DNA extraction and high‐throughput sequencing and the other half for wood physicochemical characterization (Figure A1). The tools used for cutting and splitting wood, such as handsaw blades, hammer, and chisel, as well as the workbench, were thoroughly cleaned with DNA AWAY surface decontaminant (Thermo Scientific, Waltham, MA, USA) and then wiped with 70% ethanol between uses. The scalpels and the 1/8‐in. diameter drill bits were sterilized at 180°C for 4 h.

### 2.2 DNA Extraction

To prepare samples for DNA extraction, the frozen wood was drilled immediately using a sterile 1/8‐in. diameter drill bit. The drill position was designed to ensure the samples were representative, and 15 holes were drilled per wood disc (Figure A1). The sawdust of replicate drilling samples was mixed together by shaking in centrifuge tubes to homogenize so that only the variation introduced by different DNA extraction methods would be assessed. The wood sawdust was pooled into sterile 2.0 mL tubes (filled about ¼ full) and snap‐frozen in liquid nitrogen. The tubes (prefilled with 0.1 mm glass) and spatula were precooled in liquid nitrogen before adding wood sawdust to prevent thawing. For each sample, 2–6 tubes were collected and stored at −80°C. For each method, total DNA was extracted from 400 to 500 mg (two tubes) of the pooled wood samples.

CTAB extraction buffer consisted of 2% (w/v) CTAB diluted in 100 mM Tris–HCl, 20 mM ethylenediaminetetraacetic acid (EDTA), and 1.4 M NaCl; 0.2% (v/v) β‐mercaptoethanol and 2% PVP were added before use to prevent oxidation and subsequent covalent binding of polyphenols to extracted DNA. The extraction was carried out with slight modifications following the procedure described (Allen et al. 2006). The extraction buffer was preheated in a 65°C water bath, and 1.0 mL of preheated extraction buffer was added to wood samples immediately after removing from the ultralow freezer. The mixture was vortexed for 2–5 min and incubated at 65°C for 30 min. The tubes were inverted every 5–10 min to allow mixing. After removing nonsoluble debris by centrifugation at 13,000*g* for 10 min, the homogenate was then extracted with an equal volume of phenol/chloroform/isoamyl alcohol (25:24:1). The upper aqueous phase was collected after another centrifugation at 13,000*g* for 10 min. For highly decayed wood samples, we performed the phenol/chloroform/isoamyl alcohol extraction at least twice. The DNA was precipitated in 0.6 volume of cold isopropanol and washed in 500 µL of cold 70% ethanol. The DNA pellet was air‐dried at room temperature and resuspended in 100 µL of nuclease‐free water.

The SDS extraction buffer consisted of 2% SDS (w/v) diluted in 200 mM Tris, 25 mM EDTA, and 250 mM NaCl. Additions of 0.2% (v/v) β‐mercaptoethanol and 2% PVP were made just before use to prevent oxidation and subsequent covalent bonding of polyphenols with extracted DNA. The cell lysis, phenol–chloroform extraction, DNA precipitation, wash, and hydration steps were performed as described for the CTAB method except for the following: 7 M ammonium acetate was added after incubation at 65°C and vortexed for 5–10 s to help precipitate proteins, and then nonsoluble debris was removed by centrifugation.

The Qiagen PowerLyzer PowerSoil Kit (QIAGEN LLC, Germantown, MD, USA) is comprised of lysis buffer, precipitation buffer, spin columns, wash buffer, and elution buffer. DNA was extracted according to the manufacturer protocol with slight modifications. The bead‐beating step was performed using a MoBio vortex adaptor for 2–5 min and then incubating at 65°C for 30 min. To maximize DNA yield, the column was eluted with 100 µL of elution buffer in two successive steps.

### 2.3 DNA Quantity and Quality

**Figure A2:**
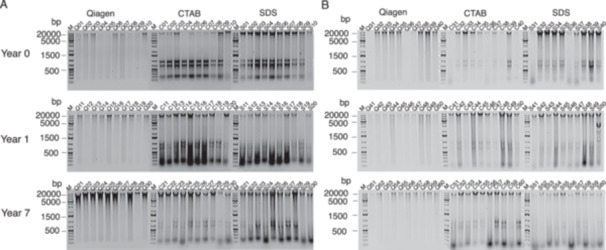
Gel electrophoresis of DNA extracted from *Betula papyrifera* (A) and *Pinus resinosa* (B) samples at different wood decay stages in the field. The samples starting with “Q” were extracted with Qiagen kits, “C” with CTAB methods, and “S” with SDS methods. The number in the sample name represents the wood disc number sampled from the field. Ten biological replicates were used for method comparisons at each sampling time for each tree host. CTAB, cetyltrimethylammonium bromide; SDS, sodium dodecyl sulfate.

DNA concentrations were measured with a Qubit 2.0 fluorometer using a Qubit dsDNA HS Assay Kit (Life Technologies, Carlsbad, CA, USA). To compare the extraction efficiency of different methods, DNA yields (ng/mg sample weight) were calculated. Absorbance ratios (A260/A280) were measured by a NanoDrop spectrophotometer, with a ratio of 1.8 indicating a pure DNA sample. Generally, for environmental DNA, this ratio is expected to range from 1.7 to 2.0. To visualize DNA integrity, the total extracted DNA was loaded on a 0.8% agarose gel. After electrophoresis at 80 V for 1 h, a digital picture was taken under UV light (Figure A2).

### 2.4 16S rRNA V6 and ITS1 Amplification by Quantitative Polymerase Chain Reaction (qPCR)

To detect if there was PCR inhibition that could affect downstream PCR or sequencing, we used qPCR to amplify bacterial 16S rRNA and fungal ITS regions using an Applied Biosystems StepOne Real‐Time PCR system (Applied Biosystems Foster City, CA, USA). The primers 926F and 1062R (Yang et al. 2015) were used to amplify the 16S rRNA V6 gene, while fungal‐specific primers NSI1 and 58A2R (Martin and Rygiewicz 2005) were used to amplify the ITS1 gene. The DNA template was adjusted to 1 ng/µL with nuclease‐free water and then diluted in a 1:10 series to 0.1 and 0.01 ng/µL. A standard curve with a slope value around −3.25 indicated that the amplification could occur among each cycle without inhibitory factors in the DNA template.

Each reaction contained 3 µL nuclease‐free water, 5 µL 2X SYBR Green Supermix (Bio‐Rad, Hercules, CA, USA), 1 µL of each primer (10 mM), and 1 µL DNA template in a final volume of 10 µL. The cycling conditions were as follows: 2 min at 98°C; 40 cycles of 15 s at 98°C, 30 s at 55°C and 1 min at 72°C; and a final extension at 72°C for 5 min. All reactions were performed in triplicate and included three nontemplate controls. The dissociation curve analyses on all plates were performed to ensure no nonspecific amplification. The amplification efficiency for 16 s rRNA V6 ranged from 89.5% to 100.5%, and for ITS1 ranged from 85.8% to 88.1%.

### 2.5 High‐Throughput Sequencing

To characterize the microbial communities in sound and decayed wood and to assess any bias introduced by DNA extraction methods, we performed amplicon sequencing on DNA extracted from the methods yielding the highest quality DNA, the Qiagen and CTAB methods. We also included a procedural CTAB blank and an extraction kit blank to identify any potential contaminating taxa during DNA extraction. It was noted that most CTAB‐extracted samples performed poorly in quality controls and needed additional purification in all CTAB samples before sequencing. The hypervariable V4 region of the 16S rRNA gene was amplified using 515f/806r barcoded primers. The ITS2 gene was amplified using 5.8SR/ITS4 barcoded primers. The library preparation and amplicon sequencing were performed by the University of Minnesota Genomics Center using the Illumina MiSeq platform with 2 × 300 bp paired‐end chemistry according to a modified dual‐indexing protocol (Gohl et al. 2016).

### 2.6 Bioinformatic Processing of Sequencing Data

Sequencing data were processed using a DADA2‐based (DADA2 version 1.16.0) bioinformatics pipeline (Callahan et al. 2016) (Schilling_Lab_pipeline). For ITS2 amplicon data, raw reads with ambiguous bases were filtered, and primers (5.8SR and ITS4) were removed using Cutadapt (version 1.18). Sequences were then filtered and trimmed using the following settings: maxEE = *c*[2, 5], truncQ = 2, maxN = 0, minLen = 50, and rm.phix = TRUE. For 16S rRNA V4 amplicon data, raw read quality was inspected first, and then sequences were filtered and trimmed using the following settings: trimLeft = *c*(19, 20), truncLen = *c*(240, 220), maxEE = *c*[2, 2], truncQ = 2, maxN = 0, and rm.phix = TRUE. All filtered and trimmed sequences were inferred using the DADA2 algorithm on pooled samples (pool = TRUE), followed by merging of paired‐end reads and removing chimeras. Using the DADA2 naïve Bayesian classifier method, the fungal taxonomic assignment was performed with the UNITE database general release dynamic files (Abarenkov et al. 2023), and the bacterial taxonomic assignment was performed with the SILVA database v138 (Quast et al. 2013). We then assigned fungi to ecological categories based on genus‐level identification using the FungalTrait database (Põlme et al. 2020).

Before downstream analyses of fungal community composition, we first noted that only 18.2% of the denoised reads were successfully merged for sound birch during the DADA2‐based bioinformatics pipeline. Therefore, after the merge step, the sound birch had fewer reads than the decayed birch (an average of 6187 vs. 27,207 reads). This low merge ratio might result from the low quality of the 3′ end in reverse reads in sound birch compared with other samples. Next, we checked that the nonfungal reads comprised 83.2% of the total DADA2 processed reads in sound birch and 48.73% in sound pine (tree host reads: 82.42% in sound birch and 46.90% sound pine), but they only comprised 6.56% of reads in decayed birch and 6.91% in decayed pine (tree host reads: 0.20% in decayed birch and 0.57% decayed pine), respectively. After removing the nonfungal reads in our data set, the sound wood had far fewer reads than decayed samples (1765 average reads in sound birch vs. 23,635 reads in decayed birch; 11,416 average reads in sound pine vs. 25,340 reads in decayed pine). To exclude the potential contaminants that might be introduced during molecular manipulation, two negative controls were included. The average three reads detected in negative controls were far fewer than in the average of 22,317 reads in wood samples. We found two contaminant taxa detected in negative controls using the “isContaminant” function from the “decontam” package in R (Davis et al. 2018), and those 2 taxa were removed from the actual wood samples. After removing samples with low sequencing reads (< 200) and amplicon sequence variants (ASVs) represented by fewer than 10 reads in any given sample, our data set of fungal communities included 1,767,510 total reads, with an average of 6786 reads per sample (range 200−27,488 reads) in sound wood and average of 24,717 reads per sample (range 8673−44,415 reads) in decayed wood. To make a comparison between decayed and sound wood samples, we opted not to rarefy the ASV table to avoid discarding additional biological information, and instead, we transformed data to relative abundance.

Before downstream analyses of bacteria community composition, chloroplast and mitochondrial reads were first measured and removed from the data set. We noted that these nonbacterial reads comprised 97.07% of the total DADA2 processed reads in sound birch and 73.31% in sound pine; however, they only represented 2.94% and 2.54% of reads in decayed birch and decayed pine, respectively. After removing those chloroplast and mitochondrial reads, the sound wood had far fewer reads than decayed samples (978 average reads in sound birch vs. 30,055 in decayed birch; 7342 average reads in sound pine vs. 34,596 in decayed pine). Next, the 8 contaminant taxa detected in negative controls were removed. We further filtered and removed samples with low sequencing reads (< 100) and removed ASVs represented by fewer than 10 reads in any given sample. After those data preprocessing steps, our data set of bacteria communities included 2,121,750 reads, with an average of 4600 reads per sample in sound wood (range 110−32,326 reads) and an average of 31,634 reads per sample in decayed wood (range 13,465−48,172 reads). It was noted that 17 of the 28 sound wood samples had a read count < 1000, while none of the 63 decayed wood samples had a read count < 1000. To make a comparison between decayed and sound wood samples, we again opted not to rarefy the ASV table, as with our fungal data.

### 2.7 Statistical Analysis

Statistical analyses and data visualizations were performed using R version 4.3.1 (R Core Team 2020). All the reported values were means ± standard errors. The analytical results were considered statistically significant with *p* < 0.05 unless otherwise noted.

We used the Friedman test, a nonparametric alternative to the one‐way repeated measures analysis of variance (ANOVA) test, to assess the effect of extraction methods on DNA yields or absorbance ratio. For this analysis, we paired the samples from the same tree host, log, and decay time across extraction methods. Therefore, the extraction method was the single independent variable in response to DNA yields or absorbance ratio. Bonferroni‐corrected pairwise Wilcoxon tests were used to compare extraction methods, again pairing samples based on all other variables. We also performed three‐way mixed ANOVA to examine the effect of extraction methods, decay time, tree hosts, and their interaction on the DNA yields. Two‐way repeated ANOVA was then performed within each tree host to confirm the interaction of extraction methods and decay time. Within each decay time and tree host, the effect of extraction methods was measured by one‐way repeated ANOVA, followed by a Bonferroni‐corrected pairwise *t*‐test to compare the methods.

The decay time effect on DNA absorbance ratio was assessed by a one‐way repeated ANOVA followed by a Bonferroni‐corrected pairwise *t*‐test. The effect of extraction methods on the copy number of fungal ITS2 or bacterial 16S rRNA V4 gene and read count of the amplicon data set was performed by paired Wilcoxon test or *t*‐test. The decay status (sound vs. decayed) effect on the read count of amplicon sequencing was examined by the Wilcoxon test.

The alpha diversity indices, including richness, Shannon, Pielou's evenness, and Faith's PD, were estimated with the “microbiome” R package (Lahti and Shetty 2017). We used the fungal or bacterial ASV table before removing the low‐abundance ASVs to generate the rarefaction curves and perform rarefaction for calculating alpha diversity. To preserve as much biological information as possible in the decayed samples, rarefaction was conducted separately for the sound and decayed samples. For fungi, the sound samples were rarefied at a depth of 1072, while the decayed samples were rarefied at 20,084. For bacteria, the sound samples were rarefied at a depth of 1350, while the decayed samples were rarefied at 21,297. The rarefaction was performed 100 times to calculate the mean value of alpha diversity. Wilcoxon tests with Bonferroni correction were used to compare alpha diversities across all possible groups of samples.

The community dissimilarity between microbial communities was measured using Bray–Curtis distances in the “vegan” R package (Oksanen et al. 2020). The overall differences in the composition of microbial communities were visualized with two‐dimensional nonmetric multidimensional scaling (NMDS) ordination plots. The effect of extraction methods, decay time, tree hosts, and their interaction were tested with permutational multivariate analyses of variance (adonis). The effect of extraction methods, decay time, and their interaction was also evaluated for each tree host independently using permutational multivariate analysis of variances (PERMANOVAs). The differences in community dispersion were tested using dispersion analysis (betadisper) and ANOVA on resulting dispersion values. For compositional analysis, we collected the core genera that appeared in at least six samples with a least 5% relative abundance (fungi) or at least six samples with a least 3% relative abundance (bacteria), and the rest of the rare genera and unidentified genera were aggregated at the phylum level. Different inclusion criteria of the core genera were chosen for fungi and bacteria to ensure that the core genera comprised approximately 50% of the community composition. We used the Wilcoxon tests with Bonferroni correction to test the difference in relative abundances of those individual core genera across all possible groups.

## 3 Results

### 3.1 DNA Yields

**Figure 1:**
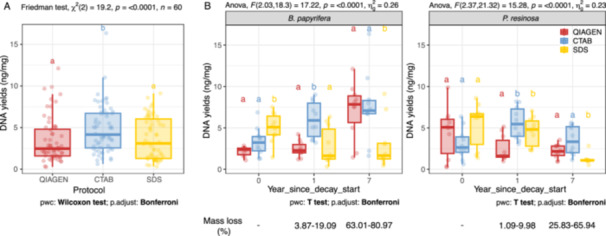
DNA extraction yields from sound or decayed wood of *Betula papyrifera* and *Pinus resinosa* using different methods. (A) Friedman test was performed to examine the effect of extraction methods on DNA yields. (B) A two‐way repeated ANOVA was performed to evaluate the effects of extraction methods, decay time, and their interactions on DNA yields within each tree host. Lowercase letters indicate significant differences between extraction methods according to the Bonferroni‐corrected pairwise paired Wilcoxon or *t*‐test comparisons. Ten biological replicates were used for method comparisons at each sampling time for each tree host. Boxes represent the 25th to 75th percentiles and display the median values (bold line in box). ANOVA, analysis of variance; CTAB, cetyltrimethylammonium bromide; SDS, sodium dodecyl sulfate.

The effect of extraction methods on the microbial genomic DNA yields was highly significant by the Friedman test (*X* ^2^(2) = 19.2, *p* = 6.77e − 05; Figure 1A). In general, the CTAB method had higher DNA yields than both the Qiagen (Bonferroni‐corrected pairwise paired Wilcoxon test, *p* = 5.13e − 04) and the SDS method (*p* = 0.015; Figure 1A). Three‐way mixed ANOVAs revealed that extraction methods and all interactions were also significant (Table S1). Two‐way repeated ANOVAs on samples within each tree host confirmed that extraction methods, decay time, and their interactions were significantly associated with the DNA yields in both birch and pine (Table S1). There was a significant effect of the extraction method on DNA yields at all sampling times for birch and at years 1 and 7 for pine (Table S1). Specifically, for sound birch wood, the DNA yields by the SDS method were significantly higher than those obtained by the Qiagen kit (Bonferroni‐corrected pairwise paired *t*‐test, *p* = 2.98e − 04) or by the CTAB method (*p* = 0.008, Figure 1B). With the development of wood decay in birch, the CTAB and Qiagen methods gradually increased their DNA yields, while the yields by SDS reduced. In contrast with sound birch wood, the DNA yields for SDS were significantly lower than for the Qiagen kit (*p* = 0.024) and CTAB (*p* = 4.65e − 04) after 7 years of decay, when birch wood had lost about 63.0%–80.9% of its original mass (decay classes IV and V; *see* Appendix 2 of Harmon et al. 2008). Red pine (Figure 1B) decayed at a slower and more variable rate than birch, with wood in decay classes II–IV by year 7. There was no significant difference between methods when extracting DNA from sound pine wood. Similarly, the DNA yields from SDS decreased along the decay gradient and were significantly lower than those obtained from Qiagen (*p* = 0.049) and CTAB (*p* = 0.004) at year 7.

### 3.2 Absorbance Ratio and Cost/Time

The mean absorbance ratio (A260/A280) for all three methods was around 1.8 for the DNA samples extracted from sound wood (Table 1). The mean absorbance ratio of decayed wood was less than 1.8, irrespective of the method used, indicating impurity in these DNA samples. The absorbance ratio was significantly affected by the extraction methods (Friedman test, *X* ^2^(2) = 27.56, *p* = 1.04e − 06). The DNA absorbance ratio of SDS extracts was significantly lower than that of Qiagen extracts (Bonferroni‐corrected pairwise paired Wilcoxon test, *p* = 3.15e − 04) and CTAB (*p* = 1.12e − 06). The decay time also had a significant effect on the absorbance ratio (repeated ANOVA, *F* = 42.35, *p* = 4.59e − 12), which was significantly higher at year 0 than year 1 (Bonferroni‐corrected paired *t*‐test, *p* = 4.86e − 08) and at year 7 (*p* = 3.63e − 08).

**Table 1:** The absorbance ratio (A260/A280), DNA color, and estimated cost and time used for one extraction by different methods for sound or decayed wood samples.

	*Betula papyrifera*			*Pinus resinosa*		
	Qiagen	CTAB	SDS	Qiagen	CTAB	SDS
Year 0						
Absorbance ratio (mean ± SE) and range	1.74 ± 0.04 1.58–1.99	1.80 ± 0.04 1.53–1.93	1.71 ± 0.04 1.37–1.78	1.81 ± 0.02 1.74–1.85	1.78 ± 0.03 1.51–2.08	NA
Color of DNA solution	Clear	Clear to light brown	Light brown	Clear	Clear to light brown	Light brown
Year 1						
Absorbance ratio (mean ± SE) and range	1.59 ± 0.06 1.44–1.93	1.60 ± 0.04 1.41–1.87	1.42 ± 0.02 1.33–1.50	1.71 ± 0.03 1.59–1.85	1.84 ± 0.06 1.42–2.02	1.56 ± 0.05 1.33–1.84
Color of DNA solution	Clear	Clear to light brown	Medium brown	Clear	Clear to light brown	Medium brown
Year 7						
Absorbance ratio (mean ± SE) and range	1.60 ± 0.05 1.39–1.79	1.51 ± 0.03 1.33–1.67	1.50 ± 0.03 1.33–1.61	NA	NA	NA
Color of DNA solution	Clear	Light brown	Dark brown	Clear	Light brown	Dark brown
Estimated extraction cost ($) and time (h) per sample	4.95–5.10 1–1.5	2.35 3–4	2.34 3–4	4.95–5.10 1–1.5	2.35 3–4	2.34 3–4
Estimated additional cost ($) for purification before sequencing	0	3.94	3.94	0	3.94	3.94

Abbreviations: CTAB, cetyltrimethylammonium bromide; NA, samples were not tested; SDS, sodium dodecyl sulfate.

The color of the DNA solution was clear for all the Qiagen samples, and it changed from clear to light brown for the CTAB samples and light brown to dark brown for the SDS samples (Table 1). The color of the DNA solution might indicate the presence of humic materials (Lakay, Botha, and Prior 2007), which generally coprecipitate with DNA due to their similar size and charge, and which inhibit downstream application. More humic materials were likely accumulating as wood progressively decomposed, which was likely explained by the darker brown color of those DNA extracts of decayed samples from CTAB or SDS methods. Those humic materials may have been efficiently removed by Qiagen columns.

The costs of CTAB and SDS methods, at the time of writing, were lower than those of the commercial kit, but the Qiagen method required the least amount of extraction time and labor with high DNA purity. It should be noted that CTAB‐extracted samples failed in quality control before HTP sequencing. An extra SPRI purification step was required before library preparation, making its total cost (extraction and additional purification) the highest at the time of writing (Table 1).

### 3.3 Amplification Efficiency for 16S rRNA and ITS regions

The DNA samples obtained by these three methods were tested for 16S rRNA V6 and ITS1 amplification using qPCR (Table 2). All DNA samples extracted using the Qiagen kit could successfully amplify the target sequence, and the standard curve slope was close to −3.25, indicating high‐purity DNA for downstream application. The CTAB and SDS methods, however, could only amplify the target sequence from sound wood. The failure to detect target sequences in decayed wood when using CTAB and SDS methods might be caused by the inhibitory compounds released from decayed wood, which was in line with the darker color and lower absorbance ratio of those extracts. Notably, the standard curve of ITS1 could not be generated from DNA extracted from either sound birch or sound pine because the threshold cycle (Ct) was too high, typically more than 30 cycles, even at the highest template loading. The primers we used for qPCR detection are fungal‐specific (Martin and Rygiewicz 2005), indicating low fungal biomass in sound wood before decay was initiated.

**Table 2:** Slope of the standard curve of 16S rRNA V6 or ITS1 amplification by qPCR.

	*Betula papyrifera*			*Pinus resinosa*		
	Qiagen	CTAB	SDS	Qiagen	CTAB	SDS
16S rRNA V6						
Year 0	−3.50 ± 0.02	−3.52 ± 0.02	−3.60 ± 0.06	−3.66 ± 0.02	−3.56 ± 0.01	−3.48 ± 0.07
Year 1	−3.50 ± 0.08	×	×	−3.41 ± 0.06	−3.37 ± 0.07	×
Year 7	−3.31 ± 0.17	×	×	−3.45 ± 0.10	×	×
ITS1						
Year 0	—	—	—	—	—	—
Year 1	−3.87 ± 0.14	×	×	−3.67 ± 0.02	−3.75 ± 0.08	×
Year 7	−4.61 ± 0.18	×	×	−3.67 ± 0.04	×	×

*Note:* “×” means the amplification failed and no standard curve could be generated; “—” means the threshold cycle (Ct) value was too high to generate a standard curve because of the low biomass in samples.

Abbreviations: CTAB, cetyltrimethylammonium bromide; qPCR, quantitative polymerase chain reaction; rRNA, ribosomal RNA; SDS, sodium dodecyl sulfate.

**Figure 2:**
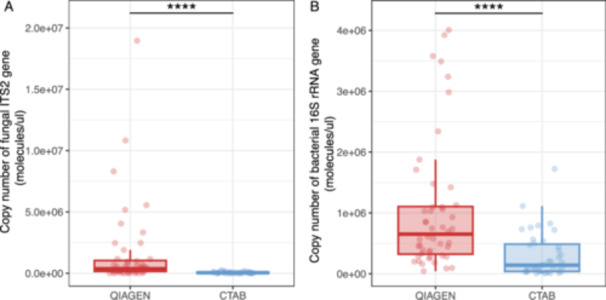
The copy number of fungal ITS2 (A) and bacterial 16S rRNA V4 (B) gene amplified from the Qiagen‐ and CTAB‐DNA extracts from sound or decayed wood of *Betula papyrifera* and *Pinus resinosa*. The stars indicate significant differences between the extraction methods according to the paired Wilcoxon comparisons. CTAB, cetyltrimethylammonium bromide; qPCR, quantitative polymerase chain reaction; rRNA, ribosomal RNA.

When we compared the gene copies of 16S rRNA V4 and ITS2 that could be amplified from Qiagen‐ and CTAB‐extracted DNA samples before amplicon sequencing, there was a substantial fraction of the CTAB samples with undetected ITS2 (13 samples) or 16S rRNA V4 (9 samples), which were all from decayed wood samples. In addition, the Qiagen had a significantly higher copy number of ITS2 (paired Wilcoxon test, *p* = 1.79e − 08) or 16S rRNA V4 (*p* = 1.81e − 08) gene compared with the CTAB method (Figure 2A,B), which further indicated the presence of inhibitors in CTAB‐extracted DNA samples. Although the DNA yields were higher using CTAB compared with Qiagen methods in some of the decayed wood samples, the increased frequency of successful amplification and higher copy number of ITS2 or 16S rRNA V4 gene detected using Qiagen extracts may have been due to the removal of the inhibitors by the Qiagen columns.

### 3.4 Extraction Effects on ITS2 Community Sequencing

**Figure 3:**
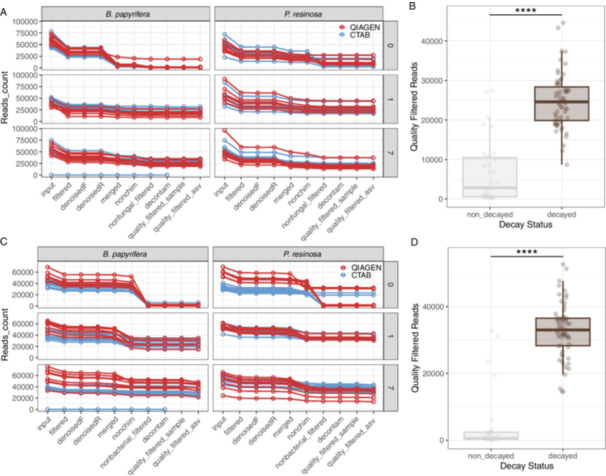
Read count summary for amplicon sequencing data of fungi (A, B) and bacteria (C, D) associated with sound or decayed *Betula papyrifera* and *Pinus resinosa* wood. The read count was tracked in DADA2‐based pipeline processing, data filtration, and quality control steps (A, C). The read counts of decayed and sound wood, after removing nonfungal or nonbacterial reads and samples with low reads (quality_filtered_sample), were compared by the Wilcoxon test (B, D). CTAB, cetyltrimethylammonium bromide.

We obtained an average fungal reads count per sample of 18,857, ranging between 201 and 44,575 (SD = 11,159). There was no significant difference in fungal reads count between Qiagen‐ and CTAB‐extracted samples by paired *t*‐test (*t*(46) = −0.811, *p* = 0.421; Figure 3A). However, the fungal reads count of sound wood was significantly lower than that of the decayed wood (Wilcoxon test, *p* = 1.17e − 11; Figure 3B), as most of the reads in sound wood were from tree host (reads of *B. papyrifera* or *P. resinosa*) and removed during nonfungal reads filtration step (Figure 3A and Table S2). We note that there has been an issue of low plant DNA discrimination from fungal sequences in another environmental study (Martin and Rygiewicz 2005).

**Figure A3:**
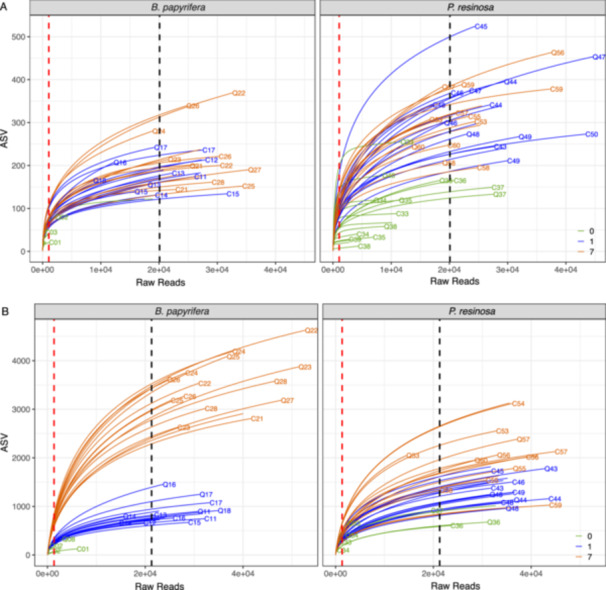
Rarefaction curves for sequenced samples, colored by decay time, for fungal (A) and bacterial (B) amplicon sequencing data of *Betula papyrifera* and *Pinus resinosa*. The rarefaction curves were generated using the ASV table before removing the low‐abundance ASVs. The red dotted line indicates the sample size (1072 for fungi and 1350 for bacteria) used for rarefaction in sound wood samples, while the black dotted line indicates the sample size (20,084 for fungi and 21,297 for bacteria) used for rarefaction in decayed wood samples. ASV, amplicon sequence variant.

Rarefaction plots showed that most of the taxa present in these samples were found at these sequencing depths (Figure A3A). Results for all alpha diversity comparisons are shown in Table S3. Samples extracted by Qiagen and CTAB were never significantly different in alpha diversity by all metrics, whether tree hosts were analyzed together or individually (Bonferroni‐corrected Wilcoxon test, *p* > 0.05). When the samples from the same tree host and decay time were used for comparison, the alpha diversities between samples extracted with Qiagen and CTAB were still not significantly different in most cases. However, birch had a significantly lower Pielou's evenness (*p* < 0.05) than pine. The alpha diversity was also significantly affected by decay time. Decayed wood samples had significantly higher richness (*p* < 0.001), Shannon (*p* < 0.01), and Faith's PD (*p* < 0.001), but a lower Pielou's evenness (*p* < 0.001) than sound wood.

**Figure 4:**
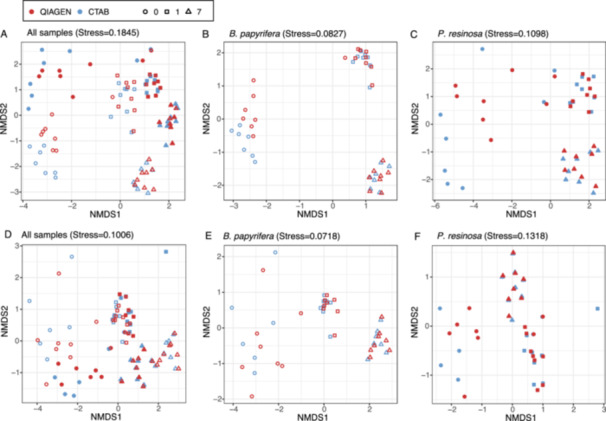
Nonmetric multidimensional scaling (NMDS) ordination plots of fungal (A–C) and bacterial (D–F) community structure along the decomposing process of *Betula papyrifera* and *Pinus resinosa*. The Bray–Curtis distance between all samples (A, D), *B. papyrifera* samples (B, E), and *P. resinosa* samples (C, F) were used for visualization. *B. papyrifera* samples and *P. resinosa* samples are represented by open and filled symbols, respectively, while colors correspond to the extraction methods and shapes correspond to decay time.

After quality filtering by removing the low‐abundance ASVs, we found 2014 fungal ASVs, with 883 ASVs in sound wood and 1855 ASVs in decayed wood. The numbers of ASVs generally ranged between 11 and 439 per sample, with an average of 180 (SD = 106) (Table S2). No pattern was apparent between Qiagen and CTAB in NMDS plots using the Bray–Curtis distance between samples (Figure 4A–C). PERMANOVA analyses found no significant difference between Qiagen and CTAB extracts (*p* > 0.9), whether samples were analyzed all together or individually within each tree host. The method‐specific interactions were also not significantly different (*p* > 0.9). However, birch and pine hosted significantly different communities (*p* = 0.001; Figure 4A). Decay time also significantly affected community composition when all samples were included (*p* = 0.001; Figure 4A). The significant effect of decay time also held true when only samples within each tree host were compared (*p* = 0.001; Figure 4B,C). The results from all statistical tests of beta diversity differences are shown in Table 3.

**Table 3:** Permutational multivariate analysis of variance results for fungal or bacterial community differences.

	Df	SumOfSqs	*R* ^2^	*F*	Pr(> *F*)^a^
*Fungi*					
All samples					
Method	1	0.155	0.004	0.493	1.000
**Year_since_decay_start**	1	3.148	0.078	9.983	0.001***
**Tree_host**	1	2.999	0.074	9.510	0.001***
**Tree_log**	14	9.068	0.224	2.054	0.001***
Method: Year_since_decay_start	1	0.116	0.003	0.368	1.000
Method: Tree_host	1	0.125	0.003	0.396	1.000
**Year_since_decay_start:Tree_host**	1	2.104	0.052	6.673	0.001***
Method: Year_since_decay_start:Tree_host	1	0.101	0.002	0.320	1.000
Residual	72	22.706	0.560		
Total	93	40.522	1.000		
Betula papyrifera					
Method	1	0.156	0.008	0.483	0.990
**Year_since_decay_start**	1	3.001	0.161	9.285	0.001***
**Tree_log**	7	4.055	0.217	1.792	0.001***
Method: Year_since_decay_start	1	0.121	0.007	0.375	1.000
Residual	35	11.312	0.607		
Total	45	18.645	1.000		
Pinus resinosa					
Method	1	0.126	0.007	0.408	0.998
**Year_since_decay_start**	1	2.275	0.121	7.388	0.001***
**Tree_log**	7	4.992	0.264	2.316	0.001***
Method: Year_since_decay_start	1	0.092	0.005	0.299	1.000
Residual	37	11.395	0.604		
Total	47	18.880	1.000		
*Bacteria*					
All samples					
Method	1	0.159	0.005	0.596	0.949
**Year_since_decay_start**	1	4.333	0.135	16.284	0.001***
**Tree_host**	1	1.466	0.046	5.509	0.001***
**Tree_log**	14	5.882	0.183	1.579	0.001***
Method: Year_since_decay_start	1	0.132	0.004	0.495	0.996
Method: Tree_host	1	0.105	0.003	0.395	1.000
**Year_since_decay_start:Tree_host**	1	1.690	0.052	6.350	0.001***
Method: Year_since_decay_start:Tree_host	1	0.091	0.003	0.341	1.000
Residual	69	18.362	0.570		
Total	90	32.220	1.000		
Betula papyrifera					
Method	1	0.146	0.009	0.535	0.933
**Year_since_decay_start**	1	3.770	0.235	13.814	0.001***
**Tree_log**	7	2.692	0.168	1.409	0.038*
Method: Year_since_decay_start	1	0.128	0.008	0.470	0.985
Residual	34	9.278	0.579		
Total	44	16.014	1.000		
Pinus resinosa					
Method	1	0.122	0.008	0.471	0.992
**Year_since_decay_start**	1	2.298	0.156	8.853	0.001***
**Tree_log**	7	3.146	0.213	1.732	0.001***
Method: Year_since_decay_start	1	0.091	0.006	0.351	0.999
Residual	35	9.084	0.616		
Total	45	14.741	1.00		

*Note:* The factors or related interactions that significantly affected fungal or bacterial community composition are shown in bold.

a Significance test: *p < 0.05, **p < 0.01, ***p < 0.001.

**Figure 5:**
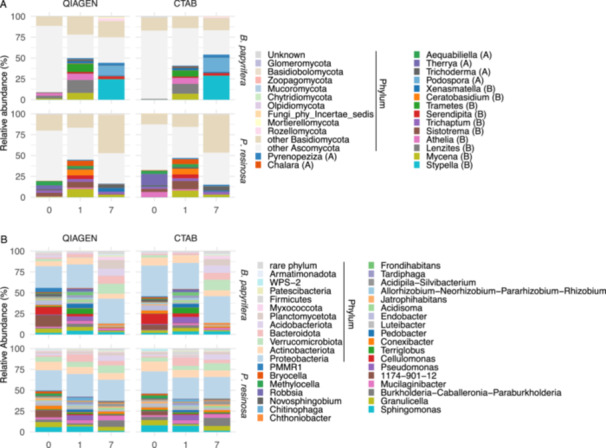
Relative abundances of fungal (A) and bacterial (B) taxa at the genus level along the decomposing process of *Betula papyrifera* and *Pinus resinosa*. For fungi, only genera with at least 5% relative abundance in at least six samples were specified, and all others, including the unidentified, were classified to the phylum level. For bacteria, only genera with at least 3% relative abundance in at least six samples were specified, and all others, including the unidentified, were classified to the phylum level. Only the top 11 bacterial phyla were shown while the other phyla were classified as rare phylum. A, Ascomycota; B, Basidiomycota; CTAB, cetyltrimethylammonium bromide.

We identified 16 core genera that occurred with a least of 5% relative abundance in at least six samples, while rare and unidentified genera were aggregated to phylum level (12). Overall, we noticed similar patterns in the relative abundance of taxa at the genus level between CTAB and Qiagen over decay time for birch or pine (Figure 5A). Of the 28 core taxa, 27 were not significantly different in abundance between Qiagen and CTAB (Bonferroni‐corrected Wilcoxon test, *p* > 0.05). The only one that showed a significant difference was classified as the rare phylum Chytridiomycota (*p* = 0.046). Within birch samples, none was significantly different in relative abundance between samples extracted by Qiagen and CTAB (*p* > 0.05). Within pine samples, the only one that had a significant difference between Qiagen and CTAB was classified as the rare phylum Olpidiomycota (*p* = 0.039). However, differences between tree hosts or across decay time were apparent. Of the tested 28 core taxa, 11 were significantly different in abundance between birch and pine (*p* < 0.05), while 25 were significantly different among decay time (Kruskal test, *p* < 0.05). The results from all statistical tests of core genera relative abundance differences are shown in Table S4.

### 3.5 Extraction Effects on 16S rRNA V4 Community Sequencing

An average bacterial reads count per sample of 24,194 was obtained with a range between 126 and 52,649 (SD = 15,568). The bacterial reads count was not significantly different between Qiagen‐ and CTAB‐extracted samples by paired *t*‐test (*t*(42) = −0.616, *p* = 0.541; Figure 3C); however, the bacterial reads count of sound wood was much significantly lower than that of the decayed wood (Wilcoxon test, *p* = 1.83e − 12; Figure 3D), as most of the reads were removed as chloroplast and mitochondrial reads in sound wood (Figure 3C and Table S2).

Rarefaction plots showed that most of the taxa present in these samples were found at these sequencing depths (Figure A3B). Results for all alpha diversity comparisons are shown in Table S3. Samples extracted by Qiagen and CTAB were not significantly different in alpha diversity by all metrics, whether tree hosts were analyzed together or individually (Bonferroni‐corrected Wilcoxon test, *p* > 0.1). When the samples from the same tree host and decay time were used for comparison, the alpha diversities between samples extracted with Qiagen and CTAB were still not significantly different in most cases. Alpha diversity (all metrics except Pielou's evenness) was significantly higher in 7‐year decayed wood than in sound wood (*p* < 0.001) and 1‐year decayed samples at year 1 (*p* < 0.001). This decay time effect on alpha diversity held true when birch samples were analyzed (*p* < 0.01). However, pine only had significantly higher richness or Faith's PD in decayed wood versus sound wood (*p* < 0.001).

After ASVs quality filtration, we found 5625 bacterial ASVs, with 1955 ASVs in sound wood and 6514 ASVs in decayed wood. The numbers of ASVs ranged from 27 to 2880 per sample with an average of 1043 (SD = 782) (Table S2). No difference was apparent between Qiagen and CTAB in NMDS plots using the Bray–Curtis distance between samples (Figure 4D–F). PERMANOVA analyses found no significant differences between Qiagen and CTAB (*p* > 0.9), whether samples were analyzed together or individually within each tree host. The method‐specific interactions were also not significantly different (*p* > 0.9). However, birch and pine communities differed significantly (*p* = 0.001; Figure 4D). Decay time was significantly associated with community differences when all samples were included (*p* = 0.001; Figure 4D). The effect of decay time was also significant within each tree host (*p* = 0.001; Figure 4E,F). The results from all statistical tests of beta diversity differences are shown in Table 3.

We identified 25 core genera that occurred with a least of 3% relative abundance in at least six samples, while the rare and unidentified genera were aggregated to phylum level (12). Overall, we noticed similar patterns in the relative abundance of taxa at the genera level between CTAB and Qiagen over decay time for birch or pine (Figure 5B). Of the 37 core taxa, none significantly differed in abundance between Qiagen and CTAB (Bonferroni‐corrected Wilcoxon test, *p* > 0.05). Within pine samples, again, none were significantly different in abundance between samples extracted by Qiagen and CTAB (*p* > 0.05). Only one genus, *Novosphingobium*, was significantly higher in CTAB‐extracted birch samples than in Qiagen‐extracted birch samples (*p* = 0.027), but its relative abundance was very low. Differences between tree hosts or across decay time were, however, apparent. Of the tested 37 core taxa, 13 were significantly different in abundance between birch and pine (*p* < 0.05), while 35 were significantly different among decay time (Kruskal test, *p* < 0.05). The results from all statistical tests of core genera relative abundance differences are shown in Table S4.

## 4 Discussion

It is known that DNA extractions, as the first step of microbial community sequencing analyses, can have a profound influence on the structure and diversity of the recovered community profiles (Bharti and Grimm 2021). One explanation could be the bias for specific taxa in the first step of DNA extraction, which involves disruption and lysis of cell walls that have their variable cell wall structure and integrity (Yuan et al. 2012). However, our study using wood as the substrate suggests that the DNA extraction methods have a major influence over DNA yields, quality, time, costs, and so forth, but have little influence on the recovered community profiles of fungi and bacteria when examining ITS2 and 16S rRNAV4 amplicon data. The microbial community structure and composition in wood samples of the same tree species and decay stage were mostly similar to each other, regardless of the DNA extraction methods we employed. Instead, there were other dissimilarities to note, including the impurity of DNA extracted by the CTAB method—this led to a significant increase in target gene amplification failure as well as a low amplification efficiency, which would either prevent successful community characterization by sequencing or would cost extra for purification before sequencing. The lack of community profile differences is reassuring when reviewing published work among studies using different extraction methods, but tactically, when processing samples, it means there is first a hurdle to clear in succeeding to amplify the ITS2 or 16S rRNA V4 gene. Together with the lack of strong bias introduced by extraction methods and DNA extraction efficiency, these findings suggest that the Qiagen kit could be considered as close to a “gold standard” for woody materials that would best suit future metagenomic efforts, if/when they become more commonplace.

The Qiagen PowerLyzer PowerSoil Kit was originally developed to extract microbial community DNA from soil samples, but it has been successfully used in a nonsoil sample, such as feces, water, food, insects, and so forth (Wesolowska‐Andersen et al. 2014; Demkina et al. 2023; Barcenilla et al. 2024; Rubin et al. 2014). With the ability to remove humic acids (Lakay, Botha, and Prior 2007), this kit proved effective in decayed wood samples where a high number of humic substances might be generated during dead biomass degradation, as well as in sound wood samples that contain high amounts of secondary metabolites, including polysaccharides and polyphenols. Drilling wood to create sawdust, especially in sound wood, releases fibers that absorb the lysis buffer and lower DNA recovery, and we found that this was not improved by liquid nitrogen grinding. Bead‐beating, a mechanical disruption of the cell wall, can improve the cell lysis efficiency and achieve high DNA yield and quality (Guo and Zhang 2013); however, it can cause shearing of nucleic acids and eventually affect sequencing library preparation. In this study, the bead‐beating time was optimized as we found vigorous beating of more than 5 min caused DNA to start to shear in wood samples. To maximize the cell lysis and DNA recovery, we optimized the manufacturer's protocol by adding a 30‐min incubation at 65°C after bead‐beating. Despite lower DNA yields using Qiagen versus the CTAB method, the copy numbers of fungal ITS2 or bacterial 16S rRNA V4 were higher in Qiagen than in CTAB‐extracted samples. In addition, some of the CTAB‐extracted DNA samples failed to detect any ITS2 or 16S rRNA V4 detection. This might be due to the presence of PCR inhibitors in CTAB‐extracted samples that could be efficiently removed using the Qiagen kit. In addition, the DNA absorbance ratio and colors also indicated the high amount of PCR inhibitors in CTAB‐extracted DNA samples, especially for decayed wood. This resulted in CTAB‐related sequencing failure or extra costs of purification to remove the PCR inhibitors before sequencing. As previously reported (Thakuria et al. 2008, Healey et al. 2014), we found that sequencing success was more dependent on DNA purity than on total DNA concentration.

In our experimental design, we also examined the community characteristics related to tree species and decay stages. In line with previous studies (Kielak et al. 2016; Lepinay et al. 2021; Baldrian et al. 2016), we observed that fungal and bacteria communities both shifted along the decay continuum, especially for fungi. We also observed that the tree host (birch vs. pine) had a significant impact on this community assembly and succession. Wood‐inhabiting fungi exhibit some preferences for tree species, most evident between deciduous and coniferous wood (Purahong et al. 2018). Those fungi latently present in the living tree have an advantage in occupying the wood territory and can transition from stress tolerance to saprotrophic strategies once a tree dies (Boddy 2001). Therefore, in addition to wood chemistry differences, wood endophytes can influence community assembly history and wood decay outcomes (Fukami et al. 2010; Parfitt et al. 2010; Song et al. 2017; Cline et al. 2018).

The study of wood‐decomposing communities, especially fungi, has gained attraction as metabarcoding and metagenome sequencing technology advances along with bioinformatic analyses. Most recent work on wood decay fungi communities has been done using the ITS region as a phylogenetic marker to classify to the species level with high accuracy (Tláskal et al. 2021b). With genome assembly and annotation of wood decay fungi continuously improving (Riley et al. 2014), shotgun sequencing is promising as more information becomes available on the reference genome database. The total DNA obtained from the woody substrate to prepare whole shotgun libraries must meet a even higher threshold to represent the whole communities in terms of taxonomic diversity and metabolic potential. Our study evaluated the DNA extraction efficiency and potential bias of different methods on amplicon sequencing of both fungi and bacterial communities associated with wood decomposition. This study will provide guidance on the evaluation and decision‐making of DNA extracts from wood samples, in terms of quantity and quality (purity and integrity), for future metagenomic studies.

## Author Contributions

**Yanmei Zhang:** conceptualization (equal), data curation (lead), formal analysis (lead), methodology (lead), resources (equal), software (lead), validation (lead), visualization (lead), writing–original draft (lead), writing–review and editing (equal). **Zewei Song:** conceptualization (supporting), data curation (supporting), resources (equal), validation (equal), writing–review and editing (supporting). **Jonathan S. Schilling:** conceptualization (lead), data curation (supporting), formal analysis (supporting), funding acquisition (lead), investigation (supporting), methodology (supporting), project administration (lead), resources (equal), software (supporting), supervision (lead), validation (equal), visualization (supporting), writing–original draft (supporting), writing–review and editing (equal).

## Ethics Statement

The authors have nothing to report.

## Conflicts of Interest

None declared.

## Supporting information

Supporting information.

## Data Availability

The data supporting this study's findings, including the demultiplexed sequence reads and associated metadata, are openly available in the Sequence Read Archive (SRA) database in NCBI: https://www.ncbi.nlm.nih.gov/bioproject/PRJNA1104661. All the raw data and scripts for sequencing data analysis, statistics, and plotting pictures are in the GitHub repository: https://github.com/Yanmei-Zhang/Zhang2024MBO.
